# Lyme Disease and Associated NMDAR Encephalitis: A Case Report and Literature Review

**DOI:** 10.3390/neurolint13040048

**Published:** 2021-09-28

**Authors:** Natalja Predkele, Jānis Mednieks

**Affiliations:** 1 Department of Neurology, Paul Stradins Clinical University Hospital, 13 Pilsonu St., LV-1002 Riga, Latvia; 2 Department of Neurology and Neurosurgery, Riga Stradins University, 16 Dzirciema St., LV-1007 Riga, Latvia; 3 Faculty of Residency, Riga Stradins University, 16 Dzirciema St., LV-1007 Riga, Latvia

**Keywords:** Lyme disease, neuroborreliosis, autoimmune encephalitis, NMDAR antibodies, NMDAR encephalitis

## Abstract

We present a case of a patient with positive N-methyl-D-aspartate receptor (NMDAR) IgG antibodies in their serum and cerebrospinal fluid (CSF) associated with neuroborreliosis. Clinically, the patient presented with symptoms of confusion, as well as behavioral and speech impairments. Regardless of antibacterial treatment, no significant improvement was achieved. Methylprednisolone provided a marked improvement in the patient’s clinical signs and CSF findings. The screening did not reveal any underlying neoplasm. Taking into account the marked clinical improvement after treatment with glucocorticosteroids, we suggest that NMDAR encephalitis is a possible autoimmune complication in neuroborreliosis patients requiring additional immunotherapy.

## 1. Introduction

Autoimmune encephalitis is a group of inflammatory diseases of the brain. The group is divided into three further groups depending on the antibody detected: The first group is classic paraneoplastic syndrome, which is associated with autoantibodies for intracellular agents, e.g., Hu, Ma1/2, Ri, Recoverin, Yo, and collapsin response mediator protein 5 (CRMP5). The second group of autoimmune encephalitis is associated with antibodies for neuronal surface antigens, e.g., N-methyl-D-aspartate receptor (NMDAR), α-amino-3-hydroxy-5-methyl-4-isoxazolepropionic acid (AMPA) receptor, leucine-rich glioma-inactivated 1 (LGI1) receptor, contactin-associated protein-like 2 (Caspr2) receptor, and the γ-aminobutyric acid (GABA) receptor. In the case of the third group, it comprises antibodies for intracellular synaptic proteins: glutamic acid decarboxylase 65 (anti-GAD65) and amphiphysin. Although there are specific manifestations associated with certain antibodies, most of the forms of autoimmune encephalitis that cause antibodies manifest many common symptoms [1].

NMDAR encephalitis is an autoimmune disease that is associated with antibodies for the GluN1 subunit of NMDAR, and it is the most common form of autoimmune encephalitis [2,3]. It usually affects young adults, and women more often than men. Clinically, NMDAR encephalitis presents with rapidly progressing behavioral impairments. In more than half of patients, prodromal symptoms have also been reported [4,5].

NMDAR encephalitis is often associated with a neoplasm, most commonly with teratomas. Cavaliere at al. found that the central nervous system infections caused by viruses from the Herpes viridae family (herpes simplex virus (HSV), varicella zoster virus (VZV), human herpes virus 6 (HHV6), and Epstein–Barr virus (EBV)) can be associated with NMDAR encephalitis [6,7,8]. Recently, an association between nonencephalitic HSV-1 infection and NMDAR encephalitis was described [9]. Cases of multiple co-infections associated with NMDAR have also been reported: HSV, Japanese encephalitis virus, *Borrelia burgdorferi*, and *Bartonella henselae* [10]. However, the exact cause cannot always be established [5].

NMDAR encephalitis in association with neuroborreliosis has also been described, for which there has only been one reported case so far [11].

In general, the outcome of NMDAR encephalitis is favorable in the case of immunotherapy [4,12].

## 2. Case Presentation

A 74-year-old man was admitted to the emergency department due to a gradual onset of back pain, myalgia, and memory impairment. Two days prior to the hospitalization, he experienced vivid dreams and behavioral and language changes. Additionally, balance impairment started five days prior to hospitalization. The patient had also experienced abdominal pain, constipation, and sleep disturbances. A day before hospitalization, the patient’s speech changed and he became confused; therefore, the emergency medical service was called.

The patient’s relatives reported weight loss that was likely due to dietary changes. The patient had been physically active until recently. His medical history included prostate cancer three years ago which was effectively treated (with no signs of metastatic process), and he been followed up by a urologist. The patient had already been vaccinated against tick-borne encephalitis.

An objective examination on the day of admission showed that the patient was confused, uncritical, agitated, and partially oriented, with a GCS of 14 points, a BP of 154/87 mmHg, a HR of 75 beats per minute, an SpO2 of 99%, and a normal body temperature.

Upon neurological examination, a full range of eyeball movements was possible, nystagmus was not observed, the pupils were symmetrical, and dysphonia, dysarthria, dysphagia and paresis were not detected. Additionally, mild asymmetry of facial mimicry muscles was observed with a droop on the left side; left-side hemihypesthesia was also observed. The periosteal tendon reflexes were symmetrical and preserved, he had a bilaterally positive Babinski reflex and mild nuchal rigidity (3 cm), and his urine retention was present.

A CT of the head performed on the emergency ward was normal. The laboratory findings indicated slightly elevated blood inflammatory markers, severe hyponatremia (124 mmol/L), CSF pleocytosis of 79 cells/uL (agranulocytes 85%), a protein level of 1.924 g/L, and a glucose level of 2.6 mmol/L (serum glucose of 5.9 mmol/L) (Table 1).

The working diagnosis on the day of hospitalization was that the patient had an electrolyte imbalance and unspecified meningoencephalitis. Immunological, viral, and bacterial tests were also performed. Empirical antiviral therapy with acyclovir and antibacterial therapy with ceftriaxone were initiated.

Day 2: An MRI of the head indicated no acute pathology, although meningioma on the right side of the forehead was revealed. Additional neurological manifestations became apparent: ataxia of the right lower extremity, as well as dysmetria of the upper extremities and a positive Romberg test.

Taking into account poor improvement with empirical therapy, a repeated lumbar puncture was performed on the third day, after which the indicators in the CSF slightly improved (Table 1) and the previous therapy was continued. The blood serum sodium level also returned to within the normal limits. Both the serum and CSF were screened for antibodies associated with autoimmune encephalitis and paraneoplastic neurologic syndromes. Serology tests for syphilis and HIV returned negative results.

Day 6: The infection work-up results were received for blood and cerebrospinal fluid analysis (Table 2), as outlined below.

Blood serum: Positive *Borrelia burgdorferi* IgM antibodies—OspC P41 and sum of points of 9 (sum of points: negative ≤ 5, equivocal = 6, positive ≥ 7; method: Immunoblot IgM antibodies, recomLine Borrelia IgM; MIKROGEN, Neuried, Germany). Positive *Borrelia burgdorferi* IgG antibodies—169 AU/mL (negative < 10 AU/mL, equivocal = 10–15 AU/mL, positive > 15 AU/mL; method: ELISA IgG antibodies, LIAISON Borrelia IgM Quant; DiaSorin, Saluggia, Italy). Positive *Borrelia burgdorferi* IgG antibodies—VIsE p41 OspC sum of points of 12 (sum of points: negative ≤ 5, equivocal = 6–7, positive ≥ 8; method: Immunoblot IgG antibodies, recomLine Borrelia IgG; MIKROGEN, Neuried, Germany).

Cerebrospinal fluid. Positive *Borrelia burgdorferi* IgM antibodies—8.9 AU/mK (negative < 2.5 AU/mL, equivocal = 2.5–3.5 AU/mL, positive > 3.5 AU/mL; method: ELISA IgM antibodies, LIAISON Borrelia IgM Quant; DiaSorin, Italy). Positive *Borrelia burgdorferi* IgG antibodies—172.6 AU/mL (negative < 4.5 AU/mL, equivocal 4.5–5.5 AU/mL, positive > 5.5 AU/mL; method: ELISA IgG antibodies, LIAISON Borrelia IgG Quant; DiaSorin, Italy). Positive *Borrelia burgdorferi* IgG antibody index (AI)—3.58 (positive AI > 1.5, normal AI = 0.7–1.3, invalid result AI < 0.7; method, *Borrelia* IgG antibody index in CSF, IgG antibodies, ELISA, AI using the Reiber’s formula).

Other CSF test results: Tick-borne encephalitis, *Ehrlichia* spp., *Anaplasma phagocytophilum*, *Neisseria meningitidis*, *Listeria monocytogenes*, *Streptococcus pneumoniae*, *Streptococcus agalactiae*, *Haemophilus influenzae*, and *Escherichia coli* K1 DNA (method: Allplex Meningitis—B Assay, Seegene, Seoul, Korea). Borrelia burgdorferi sensu lato DNA was negative (method—PCR in real time, FRT, AmliSens).

CMV DNA, HSV ½ DNA, VZV DNA, EBV, and the enterovirus DNA polymerase chain reaction (PCR) were negative (method—PCR in real time, CMV DNA quantitative, Artus CMV LC PCR Kit, QiaGen, Hilden, Germany; PCR in real time, HSV DNA quantitative, Artus HSV ½ LC PCR Kit, QiaGen, Germany; PCR in real time, VZV DNA quantitative, Artes VZV LC PCR Kit, QiaGen, Germany; PCR in real time, EBV DNA quantitative, Artus EBV LC PCR Kit, QiaGen, Germany; PCR in real time, enterovirus RNA detection, Xpert EV, Cepheid, Maurens-Scopont, France).

Elevated protein level in the CSF: Albumin (Turbidimetric)-positive—1772.4 mg/L (reference range, 100.0–300.0 mg/L) and IgG (Turbidimetric)-positive—282.5 mg/L (reference range, 6.3–33.5 mg/L). In the blood serum: Albumin (Turbidimetric)—32.2 mg/L (reference range, 35.0–52.0 mg/L) and IgG (Turbidimetric)—9.9 mg/L (reference range, 7.0–16.0 mg/L).

Day 7: Electroencephalography: No epileptic activity was observed.

Day 14: Despite antibacterial therapy, a marked clinical improvement was not achieved, and so a repeated lumbar puncture was performed. As a result, CSF pleocytosis increased to 68 cells, the predominance of agranulocytes persisted, and the protein level increased (Table 1). The results of the autoimmune encephalitis panel were obtained—the positive antibodies against NMDAR in the CSF titer were 1:10 and in blood serum titer were 1:100 (method: Cell-based assay (CBA) with an indirect immunofluorescence test (IIF); reference range, titer 1: <10 (serum, plasma; IgG) or CSF negative), (EUROIMMUN Medizinische Labordiagnostika AG, Lübeck, Germany). Anti-recoverin IgG antibodies were also found in the blood and cerebrospinal fluid. The results for the signal intensity of the EUROLineScan flatbed scanner were positive when more than 11, or very positive when more than 50; in our case, the result for the CSF was 17 and for the blood it was 13 (method: Immunoblot; EUROIMMUN Medizinische Labordiagnostika AG, Germany).

On the basis of an immunological work-up, the patient underwent therapy with methylprednisolone (1 g) intravenously over five days. An improvement in the patient’s clinical condition was observed after the first two days of treatment: Contact with the patient became more productive and his understanding, memory, and focal neurological symptoms improved.

Day 17: MOCA testing was possible, scoring 22 out of 30 points. The patient was consulted and received methylprednisolone for a total of five days, complementary to ceftriaxone. Oncological screening was additionally performed with computed tomography of the lungs and abdomen, revealing no signs of a neoplasm. The patient underwent a consultation with an ophthalmologist and urologist, but neither revealed any signs of malignancy.

Day 24: The patient showed a marked improvement and was discharged from hospital. His neurological state at discharge was oriented in time, space, and personality, with a full range of eyeball movements, symmetrical pupils, and mild asymmetry of the facial muscle group with a droop on the left side, while paresis in the limb musculature was not observed. Symmetrical periosteal tendon reflex and coordination tests were performed precisely, and sensory disturbances were no longer observed, while the meningeal signs were negative. No urinary or defecation impairment were present. A lumbar puncture was performed prior to discharge (Table 1).

The patient was discharged for outpatient treatment, with recommendations to complete a course of treatment with intravenous ceftriaxone (total, 28 days).

Day 57: No new complaints or neurological deterioration were observed. The CSF pleocytosis level was 8/uL and the protein level was 0.655 g/L. Anti-NMDAR encephalitis antibodies were negative in the cerebrospinal fluid, while anti-recoverin remained positive. A specific therapy was not recommended.

Day 340: The patient was hospitalized for a follow-up. He had been experiencing fatigue, but it had a minimal effect on his daily life. He also noticed that, after developing this condition, his memory had slightly worsened, but his memory impairment was not worsening anymore. However, he noticed that his fingertips had become less sensitive. A neurological examination revealed mild hypesthesia of both hands and feet with reduced vibration sensation, while his meningeal signs were negative. Improvement in the MOCA test was observed, with 26/30 points. The laboratory findings showed that anti-NMDAR and other autoimmune encephalitis antibodies were negative. Slightly positive anti-recoverin antibodies in the serum and CSF were found, the result was 13. The CSF test results indicated that white blood cells were absent, while the protein results were 0.52 g/L (Table 1). Nerve conduction studies showed no data of polyneuropathy. A cancer screening was repeated and no data on a possible malignant process were obtained.

## 3. Discussion

The phenomenon of autoimmune conditions being associated with an infectious disease is well known, such as in the case of Guillain–Barré syndrome and the herpes group of viruses, often associated with autoimmune neurological conditions [6,13,14]. Meanwhile, *Borrelia burgdorferi* might also be one of the autoimmunity-inducing infectious agents [15]. This possibility must be considered in Lyme disease endemic regions. Latvia is located in a Lyme borreliosis endemic zone of ticks, with approximately 200–600 people suffering from Lyme disease every year. In 2019, 25.3 per 100,000 people were infected compared to 16.8 in 2020 [16].

Neuroborreliosis has wide clinical variability, including painful radiculitis, peripheral cranial neuropathy, headaches, and possibly meningitis and encephalitis with a confused state, albeit rarely [17], which results in difficulties in distinguishing neuroborreliosis from other infectious diseases and autoimmune diseases such as autoimmune encephalitis. Herein, we described the first case in Latvia where an association between autoimmune antibodies and *B. burgdorferi* infection was observed.

Tumor and viral agents are currently known to be the most typical causes of NMDAR encephalitis, the most known tumors being teratomas [8,18], while cases of lung, breast, and testicular tumors, ovarian and thymic carcinoma, and pancreatic cancer have also been observed [4]. The study carried out by Cavaliere et al. summarized several other infectious agents associated with NMDAR encephalitis, both viruses and bacteria (EBV, HHV-6, HHV-7, VZV, influenza virus A et B, HIV, measles virus, rubella virus, mumps virus, densovirus, enterovirus, *Mycobacterium tuberculosis*, *Mycoplasma pneumonia*, *Chlamydia pneumoniae*, *Legionella pneumoniae*, *Campylobacter jejuni*, *Toxoplasma gondii*, *Angiostrongylus cantonensis*, and Japanese encephalitis virus) [7]. One of the most common viruses was HSV; one study described that approximately 27% of patients with HSV encephalitis developed autoimmune encephalitis afterward [5,6]. A case with positive autoimmune encephalitis antibodies without clinical manifestation after encephalitis, which was caused by the herpes simplex virus, was also described. The inflammation of the brain can be long-term, and it has been found that some patients can develop autoimmune antibodies without developing autoimmune encephalitis [6].

Only one case of NMDAR encephalitis caused by *Borrelia burgdorferi* was reported in 2018, and a possible pathogenesis was suggested: It is thought that Lyme disease may cause the inflammation of the nervous system, resulting in the release of NMDAR epitopes and the development of an autoimmune response [11].

The pathogenesis of NMDAR encephalitis has been extensively studied, but a clear explanation for it is still lacking. There are theories suggesting that NMDAR antigens are expressed in tumors that contain nerve cells or that antigens are secreted due to the destruction of nerve cells caused by viruses (e.g., HSV). Afterward, antigens are transported to lymph nodes, and B lymphocytes are produced. B cells, submitting to NMDAR antigens and interacting with CD4 T cells, are activated and can cross the blood–brain barrier. Activated B cells (memory cells) differentiate into anti-NMDAR-producing plasma cells [19,20]. Whether there could be a mimicry between infectious agents and NMDARs has been studied extensively, but remains to be clarified [7,20,21].

It is rather difficult to differentiate between neuroborreliosis-associated NMDAR encephalitis and positive NMDAR antibodies without a clinical manifestation, especially as the exact time of onset of both phenomena are unknown. The mean interval between infectious encephalitis and autoimmune encephalitis has been described as 25–28 days [7].

The clinical pattern of NMDAR encephalitis, in some cases, begins with prodromal symptoms, the most common of which are headaches, fever, and respiratory and gastrointestinal symptoms [12]. Mental symptoms usually appear within two weeks, such as anxiety, insomnia, delusions, and paranoia, with more than half of adult patients experiencing behavioral dysfunction. Short-term memory loss is common, as well as echolalia and mutism. In the late stage, patients may have seizures, dyskinesia, extrapyramidal symptoms, motor automatisms, autonomic instability with hyperthermia, cardiac instability, hypersalivation, bradycardia, hypertension or hypotension, incontinence, and central hypoventilation [2,4,22]. In the case of mild or incomplete clinical symptoms of the disease, mental symptoms are isolated and patients may experience seizures, dystonia, or psychiatric symptoms [22,23,24]. Cases of anti-NMDAR encephalitis presenting with a pattern of meningitis have also been reported [25,26].

Whether our patient had a clinical pattern of NMDAR encephalitis is an ambiguous question, especially when taking into account the diagnostic criteria of NMDAR encephalitis, as described by Graus et al. [18]. Our patient had a rapidly progressive clinical pattern, with memory impairment, nightmares, urine retention, and confusion, but neuroborreliosis could also present with confusion [17].

Laboratory and radiological diagnostics should also be taken into account in differential diagnostics. Considering that the patient had pleocytosis, positive neurological signs, positive *B. burgdorferi* antibodies in his blood serum, CSF, and positive AI, the diagnosis of Lyme neuroborreliosis was confirmed. In the literature, the sensitivity of AI being 80% at up to 8 weeks and 100% afterward has been described [27]. In a study of acute neuroborreliosis, an elevated mean protein level of 1.232 mg/L was presented [28]. *B. burgdorferi* DNA, sensu lato, was not revealed by PCR, which may have been due to a sensitivity of only 10–30%; early on, it can be positive, at 50%, but later declines to 13% [29].

CXCL13 may be used as an additional method in Lyme borreliosis diagnostics, as some authors suspect that CXCL13 in CSF probably has the potential to support the diagnosis of acute Lyme neuroborreliosis in patients with typical clinical symptoms and CSF pleocytosis but negative serology [30]. It is known that CXCL13 can also be elevated in other infectious, inflammatory, and neoplastic conditions. The measurement of CXCL13 for neuroborreliosis diagnostics is currently not being recommended because it has not been sufficiently studied or standardized [31].

Laboratory examinations of patients with NMDAR encephalitis show changes in cerebrospinal fluid in most patients, more commonly pleocytosis, in normal or mildly increased protein levels (median range, 0.22–1.40), and in elevated oligoclonal antibodies [3,4,32]. Pleocytosis or oligoclonal antibodies should be used to diagnose NMDAR encephalitis based on laboratory criteria [18]. Cell-based assays (CBAs) have become the gold standard for the diagnosis of NMDAR encephalitis detected by IIF, while for accurate diagnosis, tissue-based assays (TBAs) with the IIF method are recommended, followed by confirmation with CBAs [33,34]. In our case, only one CBA was used.

It is known that when tested with IIF secondary antibodies, a cross-reaction with species other than the target can occur, and this cannot be fully excluded in our case [35]. It has been mentioned in another clinical case that false-positive NMDAR antibodies may be present in cases of severe Lyme disease [36]. Studies have described that surface antibodies (detected by cell-based assays in 87.7% of cases) can be false-positive in the blood and CSF of patients with demyelinating disorders, brain infections, degenerative disorders, neurologic tumors, epilepsy, malignancies, other non-neurological diseases with inflammation, and psychiatric disorders. For example, 1.6% of the positive samples in the patient’s CSF were identified as containing Aquaporin 4 (AQP4), glycine receptor (GlyR), NMDAR/NR1, and VGKC antibodies. In a healthy group, the positive neuronal surface antibodies in the blood serum were shown to be approximately 0.23%, but in the CSF were 0.00%; however, the study included only seven healthy persons [37].

It has been described that the sensitivity of NMDAR antibodies in CSF is greater than that in serum [4]. We detected NMDAR IgG antibodies by a well-known and verified CBA test with IIF, and they were positive in both the serum and CSF, which were more indicative of autoimmune encephalitis; thus, we cannot rule out anti-NMDAR encephalitis.

The radiological examination of patients with NMDAR encephalitis has a limited sensitivity, and magnetic resonance imaging (MRI) of the head shows no change in approximately 30–50% of patients. If present, parenchymal alterations are described in FLAIR or T2 images showing an increased signal in cortical, meningeal, or basal ganglia regions [4,22]. The non-specific slowing of background activity is observed in most patients with NMDAR encephalitis upon electroencephalography (EEG), with more generalized rhythmic delta–theta activity [22] and generalized rhythmic delta activity plus rapid activity being more specific [38]. Our patient had no specific changes in his EEG or MRI results of the head.

This case is also complicated by the presence of positive intracellular anti-recoverin paraneoplastic antibodies. These antibodies are considered to be a major biomarker in cancer-associated retinopathy and small-cell lung cancer [1,39]. In this case, typical paraneoplastic syndrome–retinopathy was not detected, and no data about small cell lung cancer were obtained, even with repetitive CT scans. It has been reported that cases with positive intracellular antibodies in CSF pleocytosis are very rare, and behavioral changes are more commonly reported in patients with positive surface antibodies than intracellular antibodies [40]. Meanwhile, the data from patients with autoimmune encephalitis were summarized in one study, which described that positive anti-recoverin antibodies may clinically manifest as behavioral disorders, ataxia, and numbness [40]. The data are limited in terms of patients with positive antibodies working against cell surfaces and intracellular antibodies. There have been reports of extracellular (NMDAR) and intracellular (anti-Ri) antibodies detected simultaneously, and this patient had epileptic seizures [40]. In our case, the patient was diagnosed with positive anti-recoverin antibodies at the onset of the disease, while in a later examination, they remained positive, but only weakly, which is why paraneoplastic syndrome was suspected. The isolated use of immunoblots should be questioned regarding whether additional laboratory tests are needed; for example, a previous study described the usefulness of immunofluorescence (IF) and immunohistochemistry (IHC) onconeural antibodies tests (recoverin tests), which may be a suitable option to avoid false-positive results and unnecessary oncological screening tests [41,42]. At the moment, we believe that if we were to repeat the test against, the anti-recoverin (immunoblot) would still turn out as weakly positive, meaning that we should test it in the CSF, perform a PET scan, or carry out a control IF or IHC.

The response to immunosuppressive therapy varies, with 45% of patients without a teratoma responding well to first-line therapy, while another 48% respond poorly. However, in patients with teratoma, clinical improvement was observed in 52% after surgical removal and subsequent first-line immunotherapy. Some cases of NMDAR encephalitis without immunotherapy have also been described, and 29% of them had a poor prognosis [4]. Armangue et al. analyzed a cohort of patients with autoimmune encephalitis after HSV encephalitis, and the prognosis was worse than reported in patients with isolated NMDAR encephalitis [6]. On the contrary, in another study, the clinical outcome in patients with NMDAR encephalitis after non-herpes group CNS infection was assessed; four of the fourteen patients experienced complete or almost complete recovery, while one patient died due to an unknown reason [7]. In our case, we observed a convincing clinical improvement only after starting first-line immunotherapy with glucocorticosteroids, and the modified Rankin scale at discharge was 1.

After treatment, the patient had a noted clinical improvement, but later, he felt fatigue, which was not apparent before Lyme disease. Despite this, he was still able to carry out all of his daily activities, although sometimes had concentration difficulties, but his MOCA scale results improved a year after the acute condition. The blood and CSF analyses showed no worsening of his condition. We believe that the patient’s symptoms can also be explained by post-treatment Lyme disease syndrome (PTLDS). The prevalence of PLTDS may be at least 15% in endemic regions, and the typical symptoms are fatigue, musculoskeletal pain, cognitive dysfunction, sleep disruption, paresthesia, headaches, dizziness, and mood changes. PTLDS treatment is still a complex issue, and there are various recommendations and treatments with antibacterial medications that should be considered individually [43]. In our case, the patient had minimal symptoms and no signs of inflammation in his blood or CSF, so we decided to continue symptomatic treatment only.

## 4. Conclusions

In our case, we believe that neuroborreliosis activated certain autoimmunity mechanisms, resulting in NMDAR antibodies and clinically manifested encephalitis. Additionally, we think that the NMDAR antibodies were not only an accidental finding, but they were pathogenic. We propose that immunotherapy for patients with positive NMDAR antibodies should be considered if there is even a slight possibility of improvement.

Similar cases should be further documented and studied, preferably in case series, to improve our understanding of the pathophysiological mechanism-based relationship between infection agents and autoimmune encephalitis.

## Figures and Tables

**Table 1:** Laboratory analysis.

Time since First Hospitalization	Blood Serum				Cerebrospinal Fluid					
	Leukocytes, 10^9^/L; CRP g/L	Infective	Autoimmune Markers	Tumor Markers	Protein, g/L	Cells, uL	Agranulocytes, %	Infective	Autoimmune	Tumor Markers
Day of hospitalization	13.3; 5.14	-	-	-	1.924	79	85	-	-	-
Day 2	6.8; 37.35	-	-	-				-	-	-
Day 3		-	-	-	1.658	51	90	-	-	-
Day 6	8.4; 22.90	Positive *B. burgdorferi* AI	-	-	-	-	-	Positive *B. burgdorferi* AI	-	-
Day 14		-	Positive anti-NMDAR Abs	Positive anti-recoverin Abs	1.838	68	93	-	Positive anti-NMDAR Abs	Positive anti-recoverin Abs
Day 21		-	-	-	0.522	91	97	-	-	-
Day 57	7.3; <4.00	-	-	-	0.655	8	88	-	Negative anti-NMDAR Abs	Positive anti-recoverin Abs
Day 340	7.1; <4.00	-	Negative anti-NMDAR Abs	Weak, positive anti-recoverin Abs	0.520	0	0	-	Negative anti-NMDAR Abs	Weak, positive anti-recoverin Abs

Abbreviations: CRP = C-Reactive Protein; AI = antibody index; Abs = antibodies. Normal range: Leukocytes—4.0–10.0 10^9^/L; CRP—<4.00 g/L.

**Table 2:** Laboratory analysis—*Borrelia burgdorferi* serology.

**Time since First Hospitalization**	**Blood Serum**			**Cerebrospinal Fluid**		**Antibody Index**
	**IgM (Immunoblot)**	**IgG** **(ELISA)**	**IgG** **(Immunoblot)**	**IgM (ELISA)**	**IgG** **(ELISA)**	Positive 3.58
Day 6	Positive, 9	Positive, 169 AU/mL	Positive, 12	Positive, 8.9 AU/mL	Positive, 172.6 AU/mL	

## Data Availability

The data presented in this case report are available on request from the corresponding author.
